# Photobiomodulation Therapy (PBMT) Applied in Bone Reconstructive Surgery Using Bovine Bone Grafts: A Systematic Review

**DOI:** 10.3390/ma12244051

**Published:** 2019-12-05

**Authors:** Marcelie Priscila de Oliveira Rosso, Daniela Vieira Buchaim, Karina Torres Pomini, Bruna Botteon Della Coletta, Carlos Henrique Bertoni Reis, João Paulo Galletti Pilon, Getúlio Duarte Júnior, Rogério Leone Buchaim

**Affiliations:** 1 Department of Biological Sciences (Anatomy), Bauru School of Dentistry, University of São Paulo (USP), Alameda Dr. Octávio Pinheiro Brisolla, 9-75-Vila Universitaria, Bauru, SP 17012-901, Brazil; marcelierosso@usp.br (M.P.d.O.R.); karinatorrespomini@gmail.com (K.T.P.); brunacoletta@usp.br (B.B.D.C.); 2 Postgraduate Program in Structural and Functional Interactions in Rehabilitation, University of Marilia (UNIMAR), Avenue Hygino Muzzy Filho, 1001, Marília, SP 17525–902, Brazil; danibuchaim@usp.br (D.V.B.); carloshbreis@yahoo.com.br (C.H.B.R.); drjoaopaulopilon@gmail.com (J.P.G.P.); drgetulioduarte@hotmail.com (G.D.J.); 3 Medical School, University Center of Adamantina (UniFAI), Nove de Julho Street, 730-Centro, Adamantina, SP 17800-000, Brazil

**Keywords:** bone repair, bovine bone, low-level laser therapy, photobiomodulation therapy, tissue regeneration, xenograft

## Abstract

The use of low-level laser therapy (LLLT) with biomodulatory effects on biological tissues, currently called photobiomodulation therapy (PBMT), assists in healing and reduces inflammation. The application of biomaterials has emerged in bone reconstructive surgery, especially the use of bovine bone due to its biocompatibility. Due to the many benefits related to the use of PBMT and bovine bones, the aim of this research was to review the literature to verify the relationship between PBMT and the application of bovine bone in bone reconstruction surgeries. We chose the PubMed/MEDLINE, Web of Science, and Scopus databases for the search by matching the keywords: “Bovine bone AND low-level laser therapy”, “Bovine bone AND photobiomodulation therapy”, “Xenograft AND low-level laser therapy”, and “Xenograft AND photobiomodulation therapy”. The initial search of the three databases retrieved 240 articles, 18 of which met all inclusion criteria. In the studies concerning animals (17 in total), there was evidence of PBMT assisting in biomaterial-related conduction, formation of new bone, bone healing, immunomarker expression, increasing collagen fibers, and local inflammation reduction. However, the results disagreed with regard to the resorption of biomaterial particles. The only human study showed that PBMT with bovine bone was effective for periodontal regeneration. It was concluded that PBMT assists the process in bone reconstruction when associated with bovine bone, despite divergences between applied protocols.

## 1. Introduction

Low-level laser therapy (LLLT) has been of interest to the scientific community since 1967, when Mester et al. [1] reported its effects on hair growth in rats. It was later verified that this therapy not only stimulated cellular components, but also modulated them, establishing photobiomodulation therapy (PBMT). Further, regenerative medicine has emerged in recent decades to develop adjuvant and assistive means in pathological processes, highlighting PBMT in relation to the anti-inflammatory, anti-allergic, healing and stimulating effects of tissue growth factors [2,3,4,5].

PBMT features electromagnetic energy technology with a wavelength spectrum of 600–1100 nm, with low energy density from a constant beam (0.04–60 J/cm^2^). Laser light sources include helium–neon (HeNe) and gallium–aluminum arsenide (GaAlAs), as these sources have excellent tissue penetration [4,6]. The therapeutic effects of PBMT are based on photochemical, photoelectric and photoenergetic reactions that affect cells by altering their metabolic functions. The modulatory effect is mainly related to cytochrome C oxidase, which, via photon absorption with mitochondrial reactions, generates increased adenosine triphosphate (ATP) [7,8]. The literature points to the effects of PBMT on tissues by its modulation of biological processes for cell differentiation and proliferation [9]. Its effects are related to the repair of muscle [10], nerves [11], bone [12], and burn injuries [13], besides the reduction of inflammatory cytokines and bacterial load due to photosensitive agents and biostimulation of blood vessels [10,13,14,15]. 

Most experimental and clinical studies describe that PBMT aids in the process of tissue regeneration, demonstrating biological modulatory effects on cell differentiation [9,16,17]. Photobiomodulation of bone tissue seems to increase the results of fracture repair [18], periodontal tissue [19], implant osseointegration [20] and bone reconstruction with or without biomaterials [15,21,22,23,24,25]. Its application in clinical practice with the purpose of assisting healing after bone graft reconstruction surgery [26] is still poorly described in the scientific literature. 

Bone lesions with tissue loss can lead to changes in quality of life, especially when it concerns the face [27]. Patients requiring reconstructive surgery typically describe functional loss and physical, emotional, social and labor disturbances, as well as a financial change associated with these challenges [28]. The physiological bone remodeling process is naturally coordinated; however, imbalances may occur between bone deposition and removal. In extensive tissue defects, repair can become a challenge, requiring the use of bone grafts, implants or biomaterials. At this time, tissue engineering comes into play in helping the development of components that can lead to or assist in the reconstruction of lost tissue [29,30,31].

Bone graft material, regardless of its origin (autografts, allografts, alloplastic materials or xenografts), must have the biological, physical and chemical properties necessary for the tissue repair process. Emphasis is given to those materials that have osteointegration, osteoinduction, osteoconduction and osteogenesis capacities; however, only autologous material is capable of covering all four of these properties [32,33].

In instances of large bone loss, the need for a graft is imminent. In such a scenario, autologous bone is the first choice, but there are difficulties associated with potential morbidity of the donor site. In these cases, however, grafts tend to be absorbed before osteogenesis is complete [34]. The literature cites as necessary in the reconstruction of bone defects three simultaneous conditions: (i) osteoconductive properties; (ii) inductive properties; and (iii) the presence of bone-forming cells [35]. As an alternative material, bovine bone graft [15,20,36,37,38,39] is the most widely used due to its biocompatibility characteristics, as indicated in reconstructive areas related to traumatology, cranio-maxillary surgery, facial prosthetic rehabilitation, skeletal aging and esthetic aging [28]. Physical methods, such as low-intensity ultrasound (LIPUS) [40] and photobiomodulation therapy (PBMT), have the potential to improve the bone reconstruction process, acting or not in combination with bone grafts. 

However, gaps still exist in explaining the mechanisms of PBMT and its relationship with the widely-used bovine bone. In this context, this systematic review research was based on the PICO [41,42] strategy, P: animals or humans with bone defects, I: The use of bovine bone as a scaffold and PBMT for bone defect repair, C: comparison to non-use of these components, and O: effect on bone repair. This PICO strategy was used to verify the relationship between PBMT and the use of bovine bone in bone reconstruction surgeries in different animals, based on the results presented by scientific studies already published in the PubMed/MEDLINE, Web of Science and Scopus databases.

## 2. Materials and Methods 

This systematic review was conducted in line with the Preferred Reporting Items for Systematic Reviews and Meta-Analyses (PRISMA) checklist, as well as previously published systematic reviews [43,44].

For this study, we searched three databases, PubMed/MEDLINE, Web of Science, and Scopus, during September 2019, using the following terms as keywords: “Bovine bone AND low level laser therapy”, “Bovine bone AND photobiomodulation therapy”, “Xenograft AND low-level laser therapy” and “Xenograft AND photobiomodulation therapy”, with no restriction on publication time.

The search results were initially screened by title and then abstract to sort articles into included and excluded folders. Eligibility criteria were applied impartially by the authors regardless of the results presented by each article.

Eligibility criteria:

Inclusion criteria were:

- Use of bovine bone as a scaffold and PBMT in bone reconstructions;
- Human or animal studies;
- Publications in the English language only and which allowed full access to the text.
- Each included article should present data regarding: wavelength, output power, energy density, application protocol (points, frequency and days).

Exclusion criteria were:

- Duplicate articles;
- Excluded because title was not related to aim;
- Did not use bovine bone;
- Use of other languages (not English);
- No access;
- Literature review;
- Data absence: wavelength (nm), output power (mW); energy density (J/cm^2^); quantity of radiation.

First, we verified the works that presented titles and abstracts that related to the theme of the initial research, using the two variables: bovine bone as a scaffold and PBMT. The next step was to evaluate and restrict those articles that used bovine bone as a scaffold in animals or humans. The methodology, results and relevance were considered to list the selection of articles.

**Figure 1:**
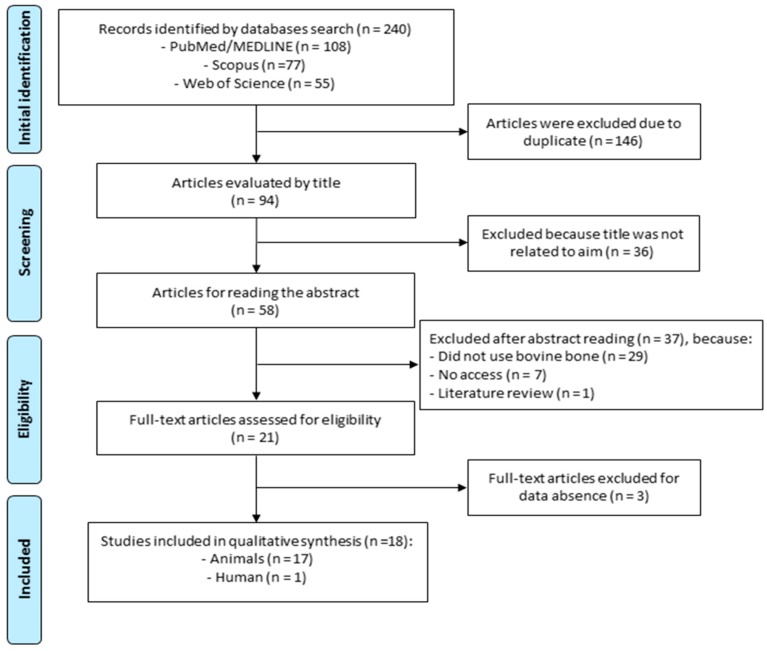
Preferred Reporting Items for Systematic Reviews and Meta-Analyses (PRISMA) flow diagram delineating the search performed in the PubMed/MEDLINE, Web of Science and Scopus databases.

Analysis and integration of reflective and consistent texts on the subject were performed. The search scheme is presented in Figure 1, according to the PRISMA flow diagram [42,44].

## 3. Results

### 3.1. Inclusion of Studies, Quality of Studies, and Test Subjects

The initial search retrieved 240 articles from the three databases, after which 146 articles were excluded because they were duplicated and 36 were excluded due to their titles being unrelated to the theme. The abstracts of 58 articles were read, resulting in the further exclusion of 37 papers as they either did not use bovine bone, did not provide access or were a literature review article, and therefore did not meet the inclusion criteria. This left 21 articles elected for full analysis. After full reading of these 21 articles, three more papers were deleted due to incomplete data. Therefore, in the end, 18 articles related to the theme were included, 17 of which were related to animals and only 1 to humans. 

Table 1 and Table 2 present the main details of the selected animal and human studies, respectively.

Evaluating the 17 articles that involved animal experiments, the total population of test subjects was 663. This total population was made up of 27 rabbits and 636 rats, divided into control groups with a total of 157 animals and intervention groups with 506 animals. The control group animals were always characterized as “empty cavity” or “clot”, while the intervention groups contained animals that underwent treatment. Nine studies used male animals [12,15,18,20,22,33,37,46,47] and seven used male and female animals [48,49,50,51,52,53,54], while only one study did not describe the gender of the subjects [45].

The periods chosen for analysis ranged from a minimum of 7 days [20] to a maximum of 90 days [15]. There appeared to be a preference seems for studies conducted up to 30 days, with 13 articles falling into this category [18,20,22,37,45,46,48,49,50,51,52,53,54], while four articles [12,15,33,47] evaluated results after this period.

Considering all the articles included in this review, the application of PBMT in bone lesions was verified in rats in 15 articles, five of which involved the calvaria [12,20,22,45,47], nine the femur [37,46,48,49,50,51,52,53,54] and one the mandibular branch [15]. The two articles employing rabbits involved the calvaria [33] and tibia [18]. The only article in humans [36] was on the alveolar bone due to periodontal disease. The use of bovine bone in its inorganic phase was observed in 10 studies [12,15,20,22,33,37,47,52,53,54] versus six using the organic phase [18,46,48,49,50,51]; only one study [45] did not offer a distinction. Bovine bone was associated with another component in 11 articles: fibrin sealant [12]; bone morphogenic proteins (BMP), collagen binder and bovine biological membrane [46,50]; hydroxyapatite/β-tricalcium phosphate (HA/βTCP) [15]; internal rigid fixation (IRF), BMP, collagen bone and decalcified cortical osseous membrane [18]; BMP and collagen gel [48]; decalcified cortical osseous membrane [51,52,54]; and collagen membrane [45].

**Figure 2:**
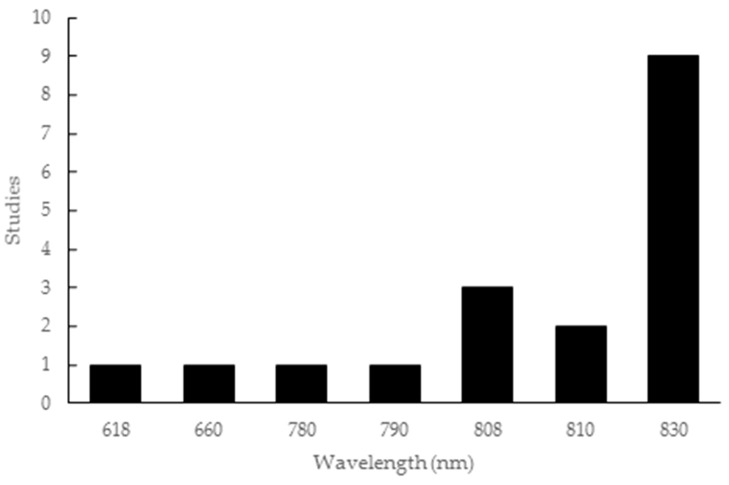
Wavelength parameters used in the articles included in this review.

The wavelength parameter employed in the studies covered a wide range of values, from 618 to 830 nm. This included one study for each of 618 nm [37], 660 nm [47], 780 nm [22] and 790 nm [18], three studies with 808 nm [15,20,45], two studies with 810 nm [33,36], and nine studies with 830 nm [12,46,48,49,50,51,52,53,54], as shown in Figure 2.

**Figure 3:**
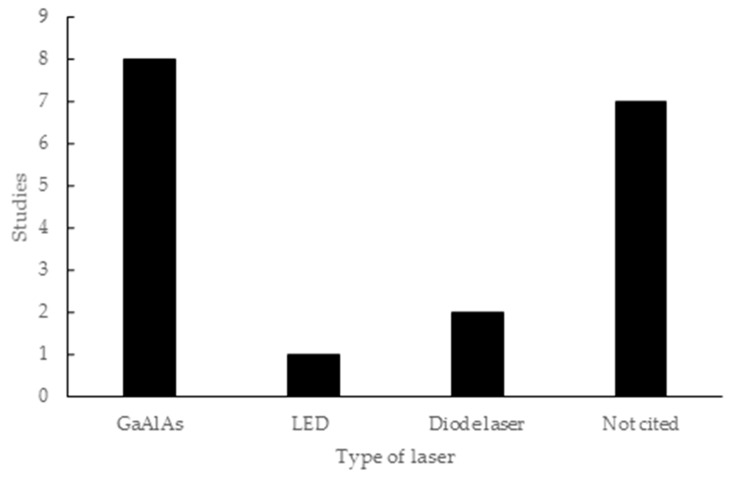
Laser types used in the articles included in this review.

Regarding the type of laser used in the studies, eight studies employed GaAlAs lasers (44.44%), one study cites the use of a light-emitting diode (LED) (5.55%), two specified the use of a diode laser (11.11%) and 7 researches did not mention the type of laser (38.88%). The seven studies that did not mention the type of laser used describe the application of the 830nm wavelength, which corresponds to the infrared range (Figure 3).

**Figure 4:**
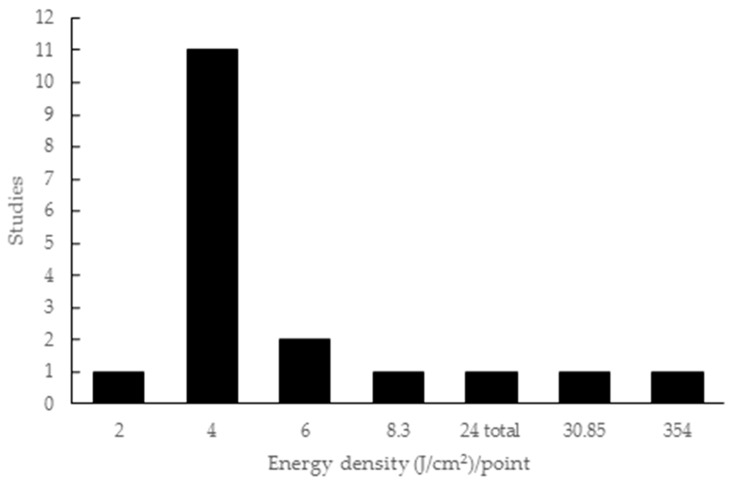
Energy density parameters used in the articles included in this review.

The energy density employed in the studies ranged from 2 to 354 J/cm^2^, with one study only citing the total energy (24 J/cm^2^) without specifying the energy per point. Eleven studies used 4 J/cm^2^ and two used 6 J/cm^2^, while energy densities of 8.3 J/cm^2^, 30.85 J/cm^2^ and 354 J/cm^2^ were applied in one study each (Figure 4).

### 3.2. Outcome Measures Used in the Included Studies

Table 3 presents the outcome measures, characteristics of the test subjects, and results obtained from the studies included in this review. Ten studies evaluated the primary outcome measure of bone density using four major methods: µCT, histological analysis of percent volume density of bone (v/v), plain X-rays and the multimodal CMS/SS OCT system. Five studies evaluated the secondary outcome measure of expression of markers, most commonly examining expression of receptor activator of nuclear factor-κB ligand (RANKL), osteoprotegerin (OPG) and receptor activator of nuclear factor-κB (RANK), through histopathological analysis, inflammatory process detection and Raman spectroscopy, and measurement of hydroxyapatite deposition.

## 4. Discussion

In recent decades, there has been a significant increase in the incidence of craniomaxillofacial and orthopedic disorders, although this has been simultaneous with remarkable progress in the development of biomaterials for reconstruction of lost bone tissue [55].

However, even though there is a wide variety of bone substitutes with satisfactory bone-filling results, histological evidence and biological behavior have only been reported for bovine bone derivatives. Thus, these xenografts have transformed reconstructive surgery and significantly improved clinical outcomes [56]. 

In addition, noninvasive, adjuvant methods in tissue regeneration have been associated with grafting techniques in an attempt to overcome some practical limits and further improve the repair results of defects filled with biomaterial. Given this context, we performed a review of the scientific literature in order to elucidate the relationship of PBMT with bovine bone when the latter is used as scaffolding for bone reconstruction.

Scientific research related to tissue engineering aims to investigate the process of bone reconstruction using scaffolds, as these are necessary as an auxiliary means for growth of new bone tissue [57]. Efforts to minimize complications and the time needed to heal by improving the process and enhancing biocompatibility has led to the emergence of PBMT-associated biomaterial application in the world literature [12,31]. Bovine bone is listed as the most frequently used type of graft in the literature for the reconstructive bone process [15,20,36,37,38,39].

Rats accounted for 95.92% of the total animals used in the articles evaluated, showing a preference for these animals in empirical study. One advantage of using rats is their easy handling due to their size, and they are generally chosen for preclinical studies in bone reconstruction biomaterial tests—being the main choice in in vivo studies in regenerative processes [58,59].

The use of male animals in nine of the studies examined suggests a preference of gender for test subjects. This decision is supported in the literature, as it avoids the possible influence of female inhibitory hormones in relation to bone tissue, in addition to the lower risk of fracture and greater bone mass [60,61].

Concerning the use of bovine bone, a preference for its inorganic phase (10 papers) was identified [12,15,20,22,33,37,47,52,53,54], although no differences in the process of bone healing when associated with a laser were reported, while six studies [18,46,48,49,50,51] used bone with an organic matrix. Bovine bone matrix has been widely used as a heterogeneous graft in orthopedic surgeries and craniofacial reconstructive procedures with satisfactory osteoconductive properties [32,33,34]. However, previous studies have shown differences between the effectiveness of inorganic and organic bovine matrices in the bone repair process. Some researches advocate for the use of inorganic material due to the absence of proteins and cells, which decreases the risk of immunogenic reactions. Further, this material provides a large amount of hydroxyapatite, which is a major component in normal bones [62]. Other researches elect to use organic material for the permanence of its protein scaffold, mainly comprised of type I collagen, which may initially favor formation of the extracellular matrix [15,63].

During this review, an array of different protocol elements was observed. A range of wavelength parameters—from 618 to 830 nm [12,37,46,48,49,50,51,52,53,54]—was used, along with variation in energy density, application time and type of laser used, even with similar types of lesions. Most articles used the infrared light spectrum [12,15,18,20,22,33,45,46,48,49,51,52,53,54], including the study on humans [36], with promotion of new (local) formations and increased protein and genes of osteoblastic factors. PBMT involves radiation from the red to infrared regions, with the latter being most cited in the literature as effective in the early stages of bone repair during the reconstruction process. This is because, at the early stages, there is a large amount of differentiating cells, and reduction of these cells at a late time of repair reduces the PBMT-related osteostimulatory potential [25,48,64]. 

Regarding the evaluation time of the experiments performed in the analyzed articles, a preference for periods up to 30 days was observed, as the literature shows more modulatory effects of PBMT during the early stages of the bone repair process. Specifically, effects such as greater proliferation of osteoblasts, collagen fibers, and mesenchymal cells, less inflammation, and greater expression of immunomarkers have been reported [12,15,18,20,37,65].

The therapeutic effects of PBMT is dependent on the mode of application, time, frequency and number of sessions of irradiation and dosing, as well as the biologically-dependent relationship of energy density and intensity. PBMT presents conflicting results in the literature, especially with regard to these modulatory effects, as the parameters (wavelength, power density, treatment dose, method and number of applications) are greatly diversified [66,67,68]. When verifying that PBMT has a major effect on mitochondria, the parameter of wavelength appears to have a major influence on the therapeutic process, with the visible (red) wavelengths activating the mitochondrial respiratory chain and the non-visible (infrared) wavelengths acting on the cell membrane. Two experiments with beneficial cellular effects of laser application can be exemplified, where greater collagen production from fibroblasts and osteoid matrix originating from osteoblasts was observed [50,66,69].

The presence of more organized collagen fibers when bovine bone grafts are associated with PBMT has been reported, relating to a biostimulatory effect on collagen production [47,48,49,50,51,52,53,54], as well as improving osteoblastic activity with the release of calcium hydroxyapatite [18,47]. This relationship with osteoblast activity seems to be related to an increase in alkaline phosphatase (ALP), bone morphogenetic protein 2 (BMP2), runt-related transcription factor 2 (Runx2) and Jagged1 differentiation genes, and osteocalcin (OCN) [15], up to a period of 30 days. Kim et al. [20] pointed to an increase of receptor activator of nuclear factor-κB ligand (RANKL), osteoprotegerin (OPG) and receptor activator of nuclear factor-κB (RANK) in the first 7 days, as already mentioned in previous studies relating bovine bones and lasers [33,70].

When using PBMT with 660 nm [47] and 618 nm [37], studies mentioned that, despite the increase of new bone, there was no resorption of bovine bone particles, while at 780 nm [22] and 808 nm [15], the biomaterial resorption occurred partially. It has been reported that osteoconductive biomaterials reduce local bone formation, which, by not being absorbed eventually, replace the new bone [63]. Oliveira et al. [15] found 60% more bone in a group without a biomaterial (control); however, computed microtomography showed that, in the groups with bovine bone, there was a greater amount of mineralized tissue. This suggests that, clinically, the use of osteoconductive biomaterials is important for maintaining morphology and function, rather than for new bone formation itself. 

Most studies used infrared spectrum wavelengths, with GaAlAs being cited in eight studies [12,15,20,22,36,45,46,47]. Seven studies [48,49,50,51,52,53,54] did not state which type of laser they used, but did describe the application of 830 nm in the infrared range. The infrared spectrum is the most widely used in reconstructive processes, as it shows less energy loss when penetrating tissues, with about 37% reaching 2 mm deep and, at larger thicknesses, the maximum loss can be as little as 162.92 mW per cm^2^ [12,71].

Bovine biomaterial is widely used and has good results in bone reconstruction processes, such as enlargement of the maxillary sinus or preparation for dental implants [72,73]. Of the articles included in this review, 17 cite positive results regarding the association of bovine bone with PBMT. However, Bosco et al. [47] concluded that PBMT stimulates bone formation regardless of the presence of biomaterial. The presence or absence of a membrane plus a biomaterial also did not seem to have an influence on the biostimulatory effects of the laser in three other studies [51,52,54]. This is in contrast to the results reported by Ghahroudi et al. [33], wherein greater bone neoformation was found when it was associated with both a biomaterial and PBMT, and the bovine bone group alone was better than a laser alone.

A critical review of the studies elected for examination showed that PBMT was associated with the promotion of new bone at lesion sites [12,15,18,20,22,33,36,37,46,47,48,51,52,53,54], increased deproteinized bovine bone (DBB) and HA/βTCP osteoconduction [15], osteoblast proteins and genes [15], increased levels of calcium hydroxyapatite (CHA) [18], metabolism and expression of immunomarkers [20] and aided the treatment of periodontal disease [36], but divergent results were found regarding particle resorption. 

A lack of persistence in the standardization of methodology employed by authors was observed, with instances of absence of important data, such as output power, energy density and application time, a pattern also observed in reviews relating PBMT to other types of lesions (such as nervous) [5]. It is extremely important to highlight the scarcity of publications addressing PBMT. This complementary treatment method is cited in the literature in association with the widely-used bovine bone scaffolds in bone reconstruction, with both having good results and clinical applicability.

## 5. Conclusions

At the end of this review, it can be verified that the data presented in recent literature shows potential to improve the bone reconstructive process using PBMT together with bovine bone as a scaffold. A variability of parameters seems to be common in studies using PBMT, as well as a lack of parameters, generating doubts regarding reproducibility and, consequently, the production of satisfactory results.

## Figures and Tables

**Table 1:** Summary of the main photobiomodulation therapy (PBMT) parameters used in animals studies.

Authors	Type of Laser (Manufacturer)	Wavelength (nm)/Spot Beam (cm^2^)	Output Power (mW)	Energy Density (J/cm^2^)	Quantity of Radiation	Bovine Bone	Therapeutic Variables	Irradiation Site (Defect)	Evaluation Time	Outcome Measures
Luca et al., 2019 [45]	GaAlAs (IRRADIA Mid-Laser Stockholm, Suécia)	808/-	450 Frequen-cy of 3800 Hz	2/1.9 J per session	4 points around the defect plus 1 central point. 17 s/point. Started IP, repeated every 48 h, until the established sacrifice day.	BBG	CM	Rat calvaria (5 mm Ø)	14, 21 and 30 days post-surgery.	By CMS/SS-OCT quantitative analysis in 30 days, BBG + PBMT with higher volume bone formation (27.11%, p ≤ 0.05). Histological analysis (by MT) shows new bone around the particles, osteoid lamellae delimited by osteoblasts.
Pomini et al., 2019 [12]	GaAlAs (Laserpulse IBRAMED, Amparo, SP, Brazil)	830/0.11	30	6	4 points in contact area, 24 s/point. Started IP, repeated every 48 h, three times a week until euthanasia.	DBBm	FS	Rat calvaria (8 mm Ø)	14 and 42 days post-surgery.	Histomorphometric analysis quantified higher bone volume density between both periods (5.6 to 10.64, p < 0.05) for the FS + DBBm + PBMT group and presence of the particles seen in the µCT. In the histological analysis (HE), the new bone started from the defect edges and there was more evidence of trabecular formation in the irradiated group FS + DBBm. Association of PBMT with xenograft and fibrin sealant had beneficial effects on bone repair.
Gerbi et al., 2018 [46]	GaAlAs (Thera Lase Surgery; DMC Equipamentos, São Carlos, SP, Brazil)	830/0.28	40	4	4 points applied in contact around the defect and was repeated every other day for 15 days, total of 7 sessions.	OmB	BMP + collagen Binder + bovine biological membrane	Rat femur (3mm Ø)	15 and 30 days post-surgery.	By histomorphometric analysis the OmB + PBMT group exhibited a larger area of newly formed bone tissue (21.11%, p < 0.05), demonstrating the efficacy of bone photobiomodulation in 30 days. Picrosirus and HE analysis show trabecular bone and complete cortical repair.
de Oliveira et al., 2018 [15]	GaAlAs (Therapy XT, DMC São Carlos-SP, Brazil)	808/0.02	100	354/ point Total energy 28 J	4 points in contact area, 10 s/point. Started IP, repeated every 48 h for 13 days, 7 sessions in total.	DBB	HA/βTCP + Teflo capsule, peripheral ring	Rat mandibular branch (Four holes of 0.5 mm Ø were made 6 mm from each other to form the edges of a square, the region was scarified).	30, 60 and 90 days post-surgery	Quantitative analysis by µCT: 90 days, higher PBMT effect on the amount of mineralized tissue associated with DBB (±63%, p ≤ 0.05) compared to non-biostimulated groups. Histomorphometry showed greater amount of new bone in the DBB + PBMT group (±25%, p ≤ 0.05). Lower amount of biomaterial in the PBMT, DBB (±30%, p ≤ 0.05). Immunohistochemi-cal analysis showed increased ALP in the irradiated DBB (45%, p < 0.05) group.
Bosco et al., 2016 [47]	GaAlAs (Bio Wave; Kondortech Equipment Ltd., São Carlos-SP, Brazil)	660/0.07	35	30.85/ point total energy of 19.44 J	8 points in contact area plus 1 central point in the scanning procedure. 72 s/point, 1 application IP.	IBBG	-	Rat calvaria (10 mm Ø)	30 and 60 days post-surgery	Histomorphometric analysis showed that the IBBG/PBMT group had the largest newly formed bone area (7.39 to 9.44, p < 0.05), and histological analysis (HE) showed a large osteoid matrix area, osteoblasts and newly formed bone around the particles at 60 days. No statistical difference for particle resorption at 30 (21.98 ± 4.10) and 60 (27.20 ± 6.39) days. PBMT can improve bone formation, but did not speed up the resorption of biomaterial particles.
Cunha et al., 2014 [22]	GaAlAs (Thera Lase DMC São Carlos-SP, Brazil)	780/0.05	100	210 6J per point	4 points in contact area plus 1 central point, 60 s/point. Application IP.	IBBG	-	Rat calvaria (5 mm Ø)	30 days post-surgery	Histomorphometric analysis showed that the group (IBBG + PBMT) presented the largest area of bone neoformation with 48.57% (p < 0.05) and smallest area of remaining particles (16.74%, p < 0.05). In the histological analysis (HE) presence of osteoid matrix with bone formation leading to the center of the defect, and parallel collagen fibers around the particles. PBMT benefited bone healing and particle resorption.
Havlucu et al., 2014 [37]	LED OsseoPulse (Biolux Research Ltd, Vancouver, Canada)	618/-	20 mW/cm^2^	24 total/ session	20 min of total application in contact with the area. Started 24 h after surgery and followed in this interval for 7, 14 and 21 days.	DBB	-	Rat femur (two defects of 3 mm Ø each)	8, 15 and 22 days post-surgery	By histomorphometric analysis in the DBB + PBMT group, all animals presented new bone tissue average >60% (p < 0.01), less inflammation (<30%, p < 0.01) and remaining particles less than 30%, p < 0.05) at 3 weeks. Histologically (HE), newly formed bone trabeculae with active osteoblasts were around the particles and reconstructed the defect.
Rasouli Ghahroudi et al., 2014 [33]	Diode laser (Giga com, China)	810/-	300	4	Applied around the surgical area IP and followed by ten applications (every other day) for the next 20 days.	IBB	-	Rabbit calvaria (Four defects 8 mm Ø each)	28 and 56 days post-surgery	A histomorphometric group of DBB + PBMT group had the highest mean of new bone formation, 41.83 and 47% at weeks 4 and 8, respectively, with statistically significant differences (p < 0.05) and an inflammation index <25% in 66.7% of the animals. Coinciding with the bone tissue presented in histology (HE), altering the auxiliary PBMT in bone healing.
Lopes et al., 2010 [18]	Diode Laser Unit, (Kondortech, São Carlos-SP, Brazil)	790/0.5	40	4/point	4 points applied transcutaneously around the area. Started IP, repeated every 48 h, per 15 days	LOBB	IRF + Biomaterial (LOBB + Collagen + BMP + Decalcified cortical osseous membrane)	Rabbit tibia (complete bone fracture, 5 mm)	30 days post-surgery	Raman spectroscopy demonstrated that biomaterial associated PBMT was effective in improving bone healing due to increased CHA levels. Highest group average IRF + biomaterial + PBMT (9316%, p = 0.05). PBMT was effective in improving bone healing.
Kim et al., 2009 [20]	GaAlAs (500DPSS, LVI Technology, Seoul, Korea)	808/0.01	96 power density of 830 mW/cm^2^	8.3/point	3 points applied in contact, 10 s/point. Started IP, repeated every 24 h, per 7 days.	DBB	-	Rat calvaria (2.7 mm Ø)	7, 14 and 21 days post-surgery.	The results of immunohistochemical analysis showed that RANKL expression (>50%, p = 0.199), OPG expression (>75%, p = 0.035) and RANK expression (<50%, p = 0.020) in the experimental group had a significant increase from 7 to 21 days. At 21 days of expression in osteoid formation and bone density in histology (Goldner’s trichrome).
Gerbi et al., 2008 [48]	Thera Lase, DMC Equipamentos, São Carlos, SP, Brazil	830/0.28	40	4/point	4 points applied in contact around the defect, begun immediately after suturing and was repeated every other day, for 15 days.	OLDBB	Biomaterial (OLDBB + collagen gel + BMP)	Rat femur (2 mm Ø)	15, 21 and 30 days post-surgery.	Qualitative analysis (HE and Sirius red) showed an increased collagen fibers (at 15 and 21 days) and amount of well-organized bone trabeculae at 30 days in laser irradiated animals. PBMT associated with biomaterial showed positive biomodulatory. effects.
Márquez Martínez et al., 2008 [49]	Thera Lase, DMC Equipamentos/São Carlos, SP, Brazil,	830/0.28	40	4/point	4 points applied in contact around the defect and was repeated every other day, for 2 weeks.	OBB	-	Rat femur (3 mm^2^ cavity)	15, 21 and 30 days post-surgery.	Qualitative analysis (HE and Picrosirus) at 30 days—higher amount of collagen fibers, evident osteoblastic activity and mature bone formation, with complete repair of the defect in group OBB + PBMT.
Pinheiro et al., 2008 [50]	DMC Equipamentos, São Carlos, SP, Brazil	830/0.28	40	4/point	4 points applied in contact around the defect and was repeated every other day, for 15 days.	OLDBB	Biomaterials (Collagen gel + BMP + bone resorbable decalcified cortical bone membrane)	Rat femur (2 mm^2^ cavity)	15, 21 and 30 days post-surgery.	Qualitative analysis (HE and Sirus red) showed that biomaterials + membrane-associated PBMT developed collagen fibers, accelerated cortical bone repair, and developed the Haversian system.
Marquez de Martinez Gerbi et al., 2003 [51]	DMC Equipamentos, São Carlos, SP, Brazil	830/0.28	40	4/point	4 points applied in contact around the defect and was repeated every other day, for 15 days, total of 7 sessions.	OBB	Decalcified cortical osseous membrane	Rat femur (3mm^2^ cavity)	15, 21 and 30 days post-surgery.	Qualitative histological analysis (HE and Picrosirus) showed positive effect of PBMT at 15 days with evident amounts of collagen fibers, osteoblastic activity and evident bone neoformation and complete repair of the defect. Positive effects of PBMT independent of organic bone or membrane.
de Assis Limeira Júnior et al., 2003 [52]	Thera Lase, DMC Equipamentos, São Carlos, SP, Brazil	830/0.28	40	4/point	4 points applied in contact around the defect and was repeated every other day for 15 days, total of 7 sessions.	IBB	Decalcified bovine cortical osseous membrane	Rat femur (3mm^2^ cavity)	15, 21 and 30 days post-surgery.	Qualitative analysis (HE and Picrosirius) showed that the level of bone neoformation did not change much until day 30 in most groups except for the PBMT + IBB + membrane group, where bone neoformation was most evident between days 21 and 30, with dense and well organized neoformed bone trabeculae and the conclusion of cortical repair.
Pinheiro et al., 2003 [53]	Thera Lase, DMC Equipamentos, São Carlos, SP, Brazil	830/0.28	40	4/point	4 points applied transcutaneouslyStarted IP, repeated every 48 h, total of 7 sessions.	IBB	-	Rat femur (3mm^2^ cavity)	15, 21 and 30 days post-surgery.	Histological qualitative analysis (HE and Picrosirius) showed that IBB + PBMT at 21 days obtained increased amount of bone neoformation and collagen fibers around the graft. At 30 days still presence of dense collagen fiber graft. PBMT had beneficial effects associated with inorganic bovine bone.
Pinheiro et al., 2003 [54]	Thera Lase, DMC Equipamentos, São Carlos, SP, Brazil	830/0.28	40	4	4 points applied transcutaneously Started IP, repeated every 48 h, total of 7 sessions.	IBB	Decalcified cortical osseous membrane	Rat femur (3mm^2^ cavity)	15, 21 and 30 days post-surgery.	Histological qualitative analysis (HE and Picrosirius) showed that at 30 days IBB + membrane + PBMT there was more pronounced, well-organized bone formation with dense trabeculae around the graft particles, the cortical repair was complete. All groups irradiated with more collagen fibers. PBMT accelerated bone repair.

Abbreviations: Immediate postoperative (IP); Bovine bone graft (BBG); Collagen membrane (CM); Diameter (Ø); Complex master slave enhanced swept source optical coherence tomography imaging instrument (CMS/SS-OCT); Masson trichrome (MT); Demineralized bovine bone matrix (DBBm); Fibrin sealant (FS); Microtomographic (µCT); Organic matrix bovine (OmB); Bone morphogenetic proteins (BMP); Deproteinized bovine bone (DBB); Hydroxyapatite/β-tricalcium phosphate (HA/βTCP); Alkaline phosphatase (ALP); Inorganic bovine bone graft (IBBG); Inorganic bovine bone (IBB); Hematoxylin and Eosin (HE); Lyophilized organic bovine bone (LOBB); Calcium hydroxyapatite (CHA); Internal Rigid Fixation (IRF); Receptor activator of nuclear factor-κB ligand (RANKL); Osteoprotegerin (OPG); Receptor activator of nuclear factor –κB (RANK); Organic lyophilized decalcified bovine bone (OLDBB); Organic Bovine Bone (OBB).

**Table 2:** Summary of the main PBMT parameters used in the human study.

Authors	Type of Laser (Manufacturer)	Wavelength (nm)/Spot Beam (cm^2^)	Output Power (mW)	Energy Density (J/cm^2^)	Quantity of Radiation	Bovine Bone	Irradiation Site	Evaluation Time	Outcome Measures
Bhardwaj, 2016 [36]	GaAlAs	810/-	100	4/point	5 min in contact with the internal margins of the flap and then 10 min without contact on the defect. Application for 5 days consecutively (outer surfaces of buccal and lingual flaps)	DBM	Treatment of intraosseous defects. Alveolar bone between 44 and 45.	30, 60 and 90 days post-surgery	By radiological measurement PBMT + DBM showed good results in clinical insertion level (CAL) gain of 4 mm, linear bone gain of 2.5 mm, bone filling of 37% and reduction of defect angle from 68° to 32°, showing a positive treatment result. Safe treatment to approach periodontal regeneration.

Abbreviations: Demineralized bone matrix (DBM).

**Table 3:** Data from included studies regarding outcome measures, subject attributes, and results.

Authors	Quantitative Analyzis	Measurements Results
Luca et al., 2019 [45]	CMS/SS-OCT	Bone volume formation: 27.11%
Pomini et al., 2019 [12]	Histomorphometric	Bone volume density: 10.64%
Gerbi et al., 2018 [46]	Histomorphometric	Bone volume density: 21.11%
de Oliveira et al., 2018 [15]	HistomorphometricµCT Immunohistochemistry	Bone volume density: ±25% Mineralized tissue: ±63% ALP (45%)
Bosco et al., 2016 [47]	Histomorphometric	Bone volume density: 9.44%
Cunha et al., 2014 [22]	Histomorphometric	Bone volume density: 48.57%
Havlucu et al., 2014 [37]	Histomorphometric Histopathological	Bone volume density: >60% Inflammation: <30%
Ghahroudi et al., 2014 [33]	Histomorphometric Histopathological	Bone volume density: 47% Inflammation: <30%
Lopes et al., 2010 [18]	Raman spectroscopy	CHA level: 9316%
Kim et al., 2009 [20]	Immunohistochemistry	RANKL (>50%), OPG (>75%), RANK (<50%)
Bhardwaj, 2016 [36]	Radiological for CAL	Linear bone gain: 2.5 mm and reduction of defect angle: 32°

Abbreviations: Complex master slave enhanced swept source optical coherence tomography imaging instrument (CMS/SS-OCT); Microtomographic (µCT); Alkaline phosphatase (ALP); Receptor activator of nuclear factor-κB ligand (RANKL); Osteoprotegerin (OPG); Receptor activator of nuclear factor-κB (RANK); Calcium hydroxyapatite (CHA); Clinical attachment level (CAL).
